# Monitoring the Degradation of Collagen Hydrogels by Collagenase *Clostridium histolyticum*

**DOI:** 10.3390/gels6040046

**Published:** 2020-11-27

**Authors:** Hon Wei Ng, Yi Zhang, Rafea Naffa, Sujay Prabakar

**Affiliations:** Leather and Shoe Research Association of New Zealand, P.O. Box 8094, Palmerston North 4472, New Zealand; honwei.ng@lasra.co.nz (H.W.N.); ethan.zhang@lasra.co.nz (Y.Z.); rafea.naffa@lasra.co.nz (R.N.)

**Keywords:** collagen, hydrogel, degradation, microbial collagenase, triple helix

## Abstract

Collagen-based hydrogels are investigated extensively in tissue engineering for their tunable physiochemical properties, biocompatibility and biodegradability. However, the effect of the integrity of the collagen triple helical structure on biodegradability is yet to be studied. In this study, we monitored the degradation of intact collagen (C-coll) and hydrolyzed collagen (D-coll) hydrogels in collagenase *Clostridium histolyticum* to understand their degradation process. Our results show that when peptides are present on the surface of the fibrils of D-coll hydrogels, cleavage of amide bonds occur at a much higher rate. The fibrillar structure of D-coll hydrogel results in a more pronounced breakdown of the gel network and dissolution of collagen peptides. The results from this work will improve the understanding of enzymatic degradation and the resulting bioabsorption of collagen materials used in drug delivery systems and scaffolds.

## 1. Introduction

Hydrogels are networks of hydrophilic polymers that are commonly used in biomedical applications including tissue engineering, wound dressings and carriers for drug delivery systems [1]. As the most abundant structural protein in animal extracellular matrices, collagen can be made into hydrogels with excellent biocompatibility and biodegradability [2]. For applications such as scaffolds and drug carriers, controllable biodegradability and bioabsorbability in vivo are crucial to the overall performance of the hydrogels [3,4]. Ideally, scaffolds implanted in vivo should be degraded and reabsorbed at an equivalent rate as the formation of the new tissues [4]. When used in the delivery of drug molecules or growth factors, the biodegradability of the hydrogels can be controlled to maintain the integrity of its network structure or allowed to degrade, depending on the desired sustained release rate [4]. It is therefore important to understand the kinetics of the biodegradation of collagen hydrogels to precisely control the performance for various biomedical applications.

To modify the degradation kinetics of collagen hydrogels, many approaches have been reported in the literature and the majority of them focus on either chemical crosslinking [5,6,7,8] or incorporating other additives to assist the formation of a stronger fibrillar network [9,10,11,12,13]. Interestingly, studies that focus on the effect of the integrity of the triple helical structure of collagen on the degradation of subsequent hydrogels are yet to be reported. In fact, the intermolecular forces between collagen triple helices have been found to influence the fibrillogenesis process both in vivo and in vitro [14,15]. Additionally, the triple helical structure is crucial in the formation of fibrillar networks and can affect the mechanical performances of collagen hydrogels [14,15,16,17]. Type I collagen triple helix consists of two identical α1 chains and a third distinct α2 chain, with any two α chains able to covalently crosslink to form a dimer, β chain [16]. The triple helical structure can be compromised when the amide bonds in the backbone break into free amino and carboxylic acid groups [14]. If an increasing amount of these functional groups are exposed on the surface of collagen molecules, the mechanism of self-assembly and consequently the mechanical performance of the hydrogel can be significantly different [18]. In the formation of gelatin hydrogels for example, instead of forming ordered fibrils like collagen, the gelation of gelatin involves a coil-helix transition accompanied by the nucleation of peptide chains to form a network [19,20,21].

Biomaterials are subjected to degradation by a range of collagenases in vivo, primarily by matrix metalloproteinases which present at low concentrations in the extracellular matrix of human tissues [22]. For modeling enzymatic degradation of biomaterials, bacterial collagenase from *Clostridium histolyticum* is commonly used [23,24] due to its comparable kinetics to the matrix metalloproteinases [25]. *Clostridium histolyticum* provides a mixture of collagenase and proteases, allowing multiple scissions in the triple helical domain of collagen molecules and ultimately reducing them into small peptides and amino acids [25,26,27].

In our study, to understand the biodegradability of hydrogels affected by the integrity of the triple helical structure of collagen, we prepared hydrogels from intact (C-coll) and hydrolyzed collagen (D-coll) and monitored their degradation using microbial collagenase from *Clostridium histolyticum*.

## 2. Results

### 2.1. Gelation of Intact Collagen (C-coll) and Hydrolysed Collagen (D-coll)

**Figure 1:**
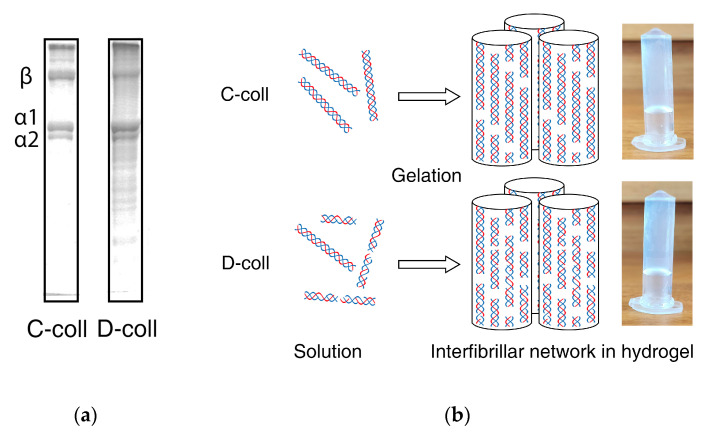
(**a**) Intact collagen (C-coll) and hydrolyzed collagen solution (D-coll) used for forming hydrogel and the corresponding sodium dodecyl sulfate–polyacrylamide gel electrophoresis (SDS–PAGE) results using 7% acrylamide gel highlighting collagen α chains and β chains, and (**b**) the schematic shows that collagen molecules assemble into the fibrillar network in the hydrogels.

Intact collagen (C-coll) was extracted from bovine hides using a conventional pepsin extraction method [28]. A modified extraction method with extended pepsin digestion during the extraction process hydrolyzed part of the intact collagen into shorter peptides to produce the hydrolyzed collagen (D-coll). Sodium dodecyl sulfate–polyacrylamide gel electrophoresis (SDS–PAGE) analysis (Figure 1) showed distinct α1, α2 and β bands in C-coll, whereas D-coll shows smearing around the α1, α2 and β bands as well as low molecular weight bands. However, when they are gelled in PBS buffer under the same pH conditions, only minor differences are visually found at the macroscopic level. Further, swelling ratio of C-coll hydrogels (Table 1) was observed to be lower than the D-coll hydrogels.

### 2.2. Degradation of Hydrogels

**Figure 2:**
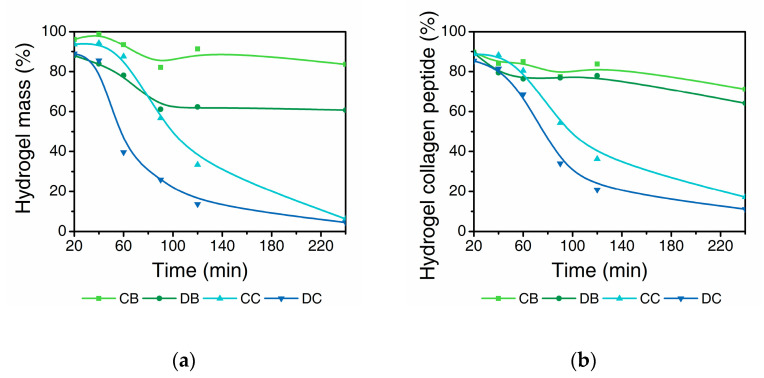
Degradation profile of collagen hydrogels. (**a**) Hydrogel mass remaining based on weight of the supernatant and (**b**) collagen peptide remaining based on hydroxyproline detected in the supernatant. CB and CC represent C-coll hydrogels in buffer and collagenase solution, respectively, while DB and DC represents D-coll hydrogels in buffer and collagenase solution, respectively. Results from one independent experiment (n = 1) are fitted with B-spline to highlight the change in the rate of degradation.

The prepared collagen hydrogels are exposed to the solution of collagenase at 37 °C to study the degradation over time for 240 min. The degradation profiles are expressed as: (a) the total mass of hydrogel, measured by the weight of supernatant and (b) mass of total collagen peptides measured by the hydroxyproline content in the supernatant. C-coll and D-coll hydrogels in Tris buffer at pH 7.4 (CB and DB) showed an initial mass loss followed by a plateau after 90 min (Figure 2a). In contrast, the hydrogels exposed to collagenase in the same buffer (CC and DC) show a rapid decrease in mass to <10%. Interestingly, CB shows a lower mass loss of 15% compared to DB at 40% at the end of the 240 min period. The difference in the mass loss of CC and DC samples increased after 60–90 min but the curves merge at the end, following a sigmoid trend with a notable lag for approximately 20 min.

The corresponding results of collagen peptides remaining insoluble in the hydrogels show a similar trend (Figure 2b). However, the difference between CB and DB is found to be much smaller compared to the mass loss displayed in Figure 2a. Similarly, this difference is less distinct when comparing CC and DC. For CB and DB, the remaining collagen peptides are 70–80%, much higher than that of CC and DC samples at around 20%, showing the effect of the collagenase.

**Figure 3:**
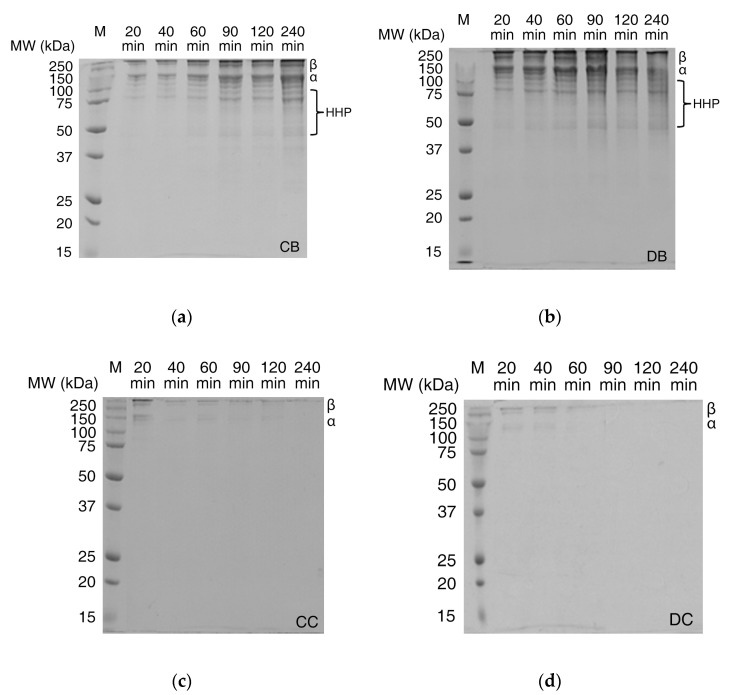
SDS–PAGE of supernatants using 12% acrylamide gel of (**a**) C-coll hydrogel in Tris buffer, pH 7.4 (CB); (**b**) D-coll hydrogel in Tris buffer, pH 7.4 (DB); (**c**) C-coll hydrogel in collagenase solution in Tris buffer, pH 7.4 (CC) and (**d**) D-coll hydrogel in collagenase solution in Tris buffer, pH 7.4 (DC). The lane of protein marker is labelled as M and shows the corresponding molecular weights. The collagen bands of α chains, β chains and high molecular weight hydrolyzed peptides (HHP).

Further, SDS–PAGE analyses of the supernatants were performed to characterize the molecular weight distribution of intact collagen peptides (α chains and β chains) and high molecular weight hydrolyzed peptides (HHP, MW = 50–100 kDa) in solutions (Figure 3). Distinct α and β bands as well as HHP bands were detected for hydrogels in buffer (CB and DB). Results from Figure 3a,b show the intensity of bands increasing with time, as more collagen and collagen peptides dissolve into the supernatant. Collagen in D-coll hydrogels (Figure 3b) were also found to dissolute more rapidly compared to C-coll hydrogels (Figure 3a) complementing the results shown in Figure 2b.

Enzymatically degraded hydrogels (CC and DC in Figure 3c,d), both C-coll and D-coll hydrogels, show weak α and β bands, gradually disappearing after 60 min. However, HHP bands were not observed in the SDS–PAGE, which contrasts with results from the hydroxyproline assay of the supernatant (Figure 2b). Hence, size-exclusion chromatography (SEC) was performed to elucidate the size distribution of degradation products, especially for low molecular weight hydrolyzed peptides (LHP) and amino acids (AA).

**Figure 4:**
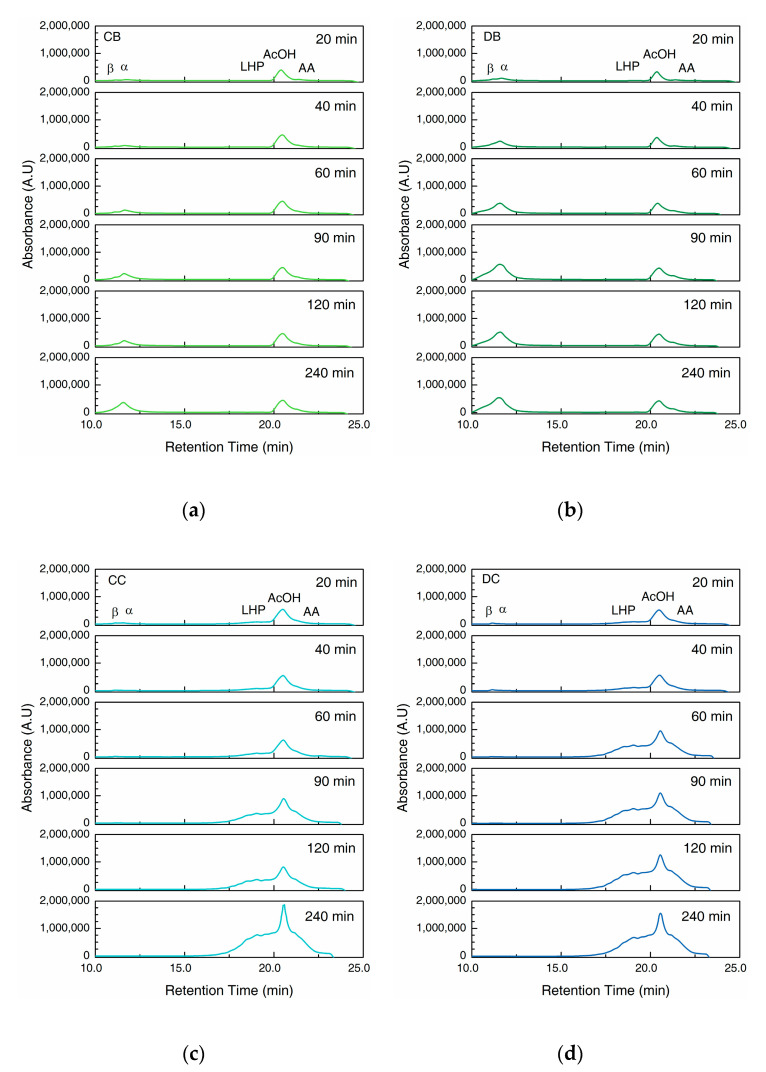
Chromatograms of size-exclusion chromatography (SEC) of supernatants collected at a wavelength of 210 nm. (**a**) C-coll hydrogel in Tris buffer, pH 7.4 (CB); (**b**) D-coll hydrogel in Tris buffer, pH 7.4 (DB); (**c**) C-coll hydrogel in collagenase solution in Tris buffer, pH 7.4 (CC) and (**d**) D-coll hydrogel in collagenase solution in Tris buffer, pH 7.4 (DC). Peaks are labelled as: β = collagen β chains; α = collagen α chains; LHP = low molecular weight hydrolyzed peptides; AcOH = acetic acid; AA = amino acids.

In the first part of the chromatogram (Figure 4), we observe two overlapping peaks from intact collagen peptides at the retention time of 10.0 to 13.0 min. The shoulder on the left side is assigned to the β band as seen on the SDS–PAGE gels, whereas the peak on its right can be assigned to α band. The second part ranging from 16.0 to 23.0 min displays the LHP (16.0 to 20.0 min) and AA (21.0 to 23.0 min), separated by the absorption peak of acetic acid (AcOH, 20.0 to 21.0 min) (Figure S1).

The hydrogels in buffer solution (CB and DB, Figure 4a,b) show the prominent α and β peaks with the intensities increasing constantly until the end of the 240 min period, which agrees with results from the hydroxyproline assay (Figure 2b) and SDS–PAGE of the supernatant (Figure 3a,b). The LHP and AA peaks are hardly visible in CB and DB, showing the negligible degradation without the collagenase.

However, when exposed to collagenase (CC and DC, Figure 4c,d), the α and β peaks are visible at the beginning of the experiment, but diminish after 90 min. In contrast, LHP and AA peaks show a prominent increase with time. DC shows a more rapid increase compared to CC within the first 120 min (Figure 4c,d), however, after 240 min, the difference becomes minimal.

**Figure 5:**
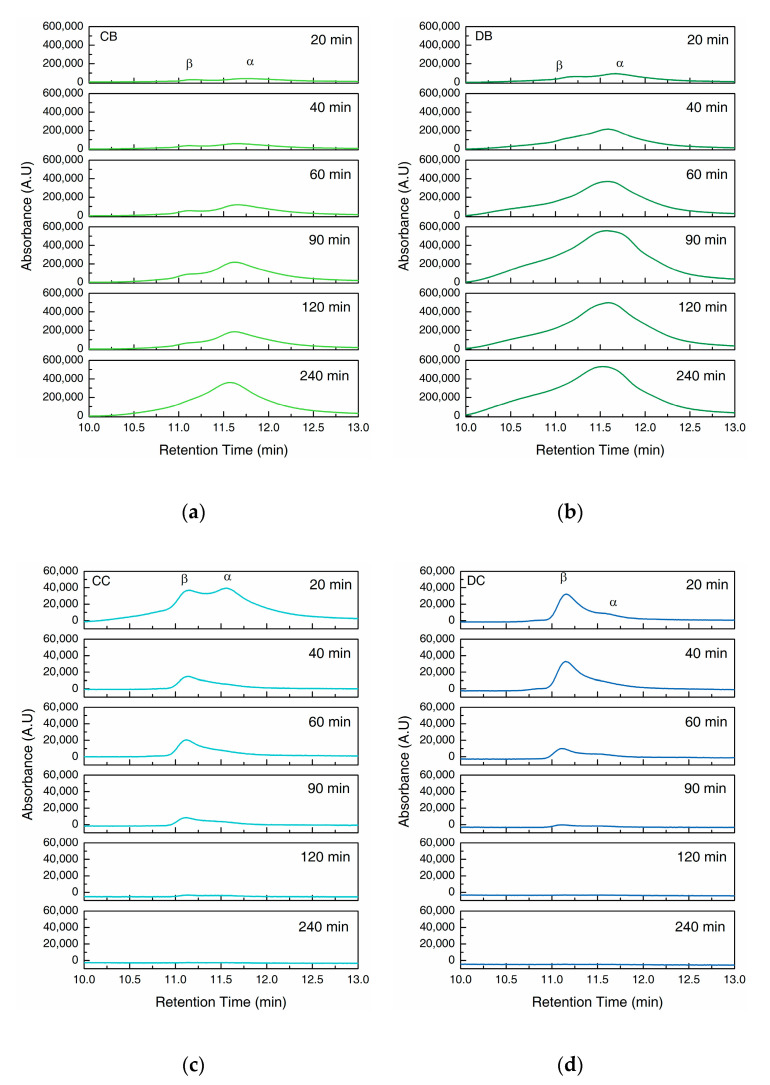
Chromatograms of size-exclusion chromatography (SEC) of supernatants collected at a wavelength of 210 nm. (**a**) C-coll hydrogel in Tris buffer, pH 7.4 (CB); (**b**) D-coll hydrogel in Tris buffer, pH 7.4 (DB); (**c**) C-coll hydrogel in collagenase solution in Tris buffer, pH 7.4 (CC) and (**d**) D-coll hydrogel in collagenase solution in Tris buffer, pH 7.4 (DC). Peaks are labelled as: β = collagen β chains; α = collagen α chains.

A closer look at the α and β peaks (Figure 5) in the chromatograms of CB and DB indicate the increasing concentration of intact collagen peptides in supernatant with time. However, β peak was overshadowed by the rapid growth of α peak, indicating a relatively faster increase in the concentration of α chains compared to the β chains in the supernatant. During enzymatic degradation, CC showed α and β peaks with similar intensity at 20 min. The intensity of α peak then decreases rapidly after 40 min, whereas β peak decreases gradually. In contrast, DC showed a much lower α peak compare to β peak at 20 min which diminishes over time.

## 3. Discussion

### 3.1. Gelation of Intact Collagen (C-coll) and Hydrolysed Collagen (D-coll)

The gelation of collagen is known to be driven by the interactions between the side chains of collagen peptides [14]. For intact collagen (C-coll), the gelation follows a similar mechanism to fibrillogenesis found in vivo, by which collagen molecules pack closely alongside each other to form dense fibrillar networks. In the case of hydrolyzed collagen (D-coll), due to an increase in amino and carboxylic acid groups, more hydrogen bonding interactions can happen between the collagen and water. This could explain the higher swelling ratio in D-coll hydrogels (778%) compared to the C-coll hydrogel (724%).

### 3.2. Degradation of Hydrogels

In a buffer solution without collagenase, the loss of bulk water in addition to the dissolution of the collagen molecules from the fibrillar network result in the mass loss of the hydrogels, as shown in Figure 2a. In addition, the concentration of the collagen peptide and the hydroxyproline content in the supernatant was found to increase over time (Figure 2 and Figure 3), accompanied by a decrease in the molecular weight as seen from the SEC results (Figure 4 and Figure 5).

In buffer, the D-coll hydrogels (DB) show a much higher mass loss compared to C-coll hydrogel (CB) of around 20–25% throughout the 240 min. However, only a slight difference in the dissolution of collagen peptide was found between CB and DB of around 5%, according to the hydroxyproline content in the supernatant. We speculate that, D-coll, due to its loss of triple helical structure, produces hydrogels that disintegrate more than hydrogels produced by C-coll, causing the increasing loss of bulk water. This effect is a major contribution to the mass loss of the hydrogels. At the same time, D-coll hydrogels have a comparatively larger intermolecular distance in the fibrils, leading to a higher rate of dissolution, supported by previous studies in hydrogels made from pronase-degraded collagen [15]. Additionally, the stronger hydrogen bonding interaction between D-coll with the solvent as stated previously, could accelerate the dissolution of collagen peptides into the supernatant. The SEC results of CB and DB agree with the increasing trend in hydroxyproline content of the supernatant. In addition, the low intensities of LHP and AA peaks throughout the experiment confirm the minimal degradation effect in the absence of collagenase.

When the collagenase is added, the cleavage of amide bonds in the collagen peptide plays an important role in the degradation process of the hydrogels. Sigmoid curves with a notable lag were observed in CC and DC and could be caused by the diffusion of the enzyme through the bulk of the hydrogels, similar to the previous report by Tzafriri et al. [29] After the lag, D-coll hydrogel was found to degrade more rapidly than its C-coll counterpart (CC and DC curve in Figure 2). The intact triple helical region of collagen molecules resist cleavage by non-specific proteases in the bacterial collagenase used in this study [30]. However, D-coll contains segments of collagen peptides which are vulnerable to proteases [30]. This results in a much higher rate of the degradation of D-coll into LHP and AA than C-coll, as shown in the SEC chromatograms (Figure 4c,d). It is also worthy of mention that in both CC and DC, the β chains were less susceptible to degradation by collagenase, due to intrinsic crosslinks that exist in the dimer and suppress the enzymatic degradation [31].

## 4. Conclusions

In this study, we monitored the degradation of collagen hydrogels in buffer and in collagenase *Clostridium histolyticum*. Segments of collagen peptides in D-coll hydrogels resulted in a much higher rate of amide bond cleavage. We postulate that the fibrillar structure of D-coll hydrogel also caused a more pronounced breakdown of the gel network and dissolution of collagen peptides. Our study highlights the important role of intact triple helical structure on the biodegradability of collagen hydrogels. The results from this work will improve the understanding of enzymatic degradability of collagen materials used for drug delivery systems and scaffolds and the resulting bioabsorption of such materials.

## 5. Materials and Methods

### 5.1. Collagen Extraction

Collagen was extracted from bovine lime split according to a procedure by Li et al. [28]. Briefly, pieces from lime split bovine hide were frozen at −20 °C before they were used for collagen extraction. All the collagen extraction steps were carried out at 4 °C. Frozen pieces were soaked in 0.5 M acetic acid solution with 2.0 wt% pepsin (2336 FIP.U/mg, BIP1008, Apollo Scientific, Stockport, United Kingdom) added. For intact collagen (C-coll), the viscous collagen extract was collected after 24 h. The hydrolyzed collagen (D-coll) was collected after 48 h. Insoluble pieces were removed using centrifugation at 15,317× *g* for 20 min. The collagen molecules were then salted out at 2.0 M NaCl concentration and subsequently collected. The precipitated collagen pellet was dissolved in a small volume of 0.5 M acetic acid to give a viscous solution and it was dialyzed against 0.05 M acetic acid three times for 24 h to remove the NaCl. The collagen solution was then freeze-dried and stored.

### 5.2. Hydrogel Preparation

Freeze-dried collagen was swollen in 0.05 M acetic acid at 4 °C overnight to produce a 1% (*w/v*) suspension. The resulting suspension was homogenized on ice for 5 min at 15,317× *g*. A quantity of 20 mL of 1% (*w/v*) collagen solution was added with 10 mL of water followed by 5 mL of 10× phosphate buffered saline. Under stirring, 0.5 M NaOH was added dropwise to raise the pH of the mixture to pH 7.4. The solution was diluted with water to a final collagen concentration of 0.4% (*w/v*) or 4 mg/mL. A volume of 1.0 mL of the solution was aliquoted into 2 mL Eppendorf tubes and subsequently incubated at 37 °C for 24 h for gelation.

### 5.3. Swelling Ratio

Freeze-dried hydrogels were soaked in phosphate buffered saline at room temperature overnight. The hydrogels were then removed from the solution, gently dabbed using filter paper and weighted. The swelling ratio of the samples was calculated using Equation (1):

Swelling Ratio (%) = (W_wet_ − W_dry_)/W_dry_ × 100%, (1)

where W_dry_ is the weight of the freeze-dried hydrogels and W_wet_ is the weight of the samples after soaking overnight.

### 5.4. Enzymatic Degradation

The enzymatic degradability of the collagen hydrogels was evaluated by *Clostridium histolyticum* collagenase. Enzyme solution was made by dissolving collagenase from *Clostridium histolyticum* (380 U/mg, C9891, Sigma-Aldrich, St. Louis, MO, USA) in 50 mM Tris/HCl buffer solution containing 5 mM CaCl_2_ (pH 7.4) to a concentration of 30 U/mL. 0.3 mL of this enzyme solution was added to 1.0 mL of collagen hydrogel in 2 mL Eppendorf tubes at 37 °C. As a control, 0.3 mL of Tris buffer (50 mM Tris/HCl buffer solution containing 5 mM CaCl_2_ (pH 7.4)) was added to 1.0 mL hydrogels. At a given time interval (20–240 min), the mixture was centrifuged at 15,317 rpm for 10 min, followed by separating the supernatant for freeze drying and rehydrating in 0.5 M acetic acid for further analysis.

### 5.5. Hydroxyproline Assay

The peptide concentration in the collected supernatants was determined using hydroxyproline assay as previously reported [32]. Briefly, supernatant of the collagenase digestion solution at each time point was hydrolyzed at a final concentration of 6 M hydrochloric acid at 105 °C for 24 h. The samples were then vacuum dried and rehydrated to 0.4 mL volume. An amount of 0.2 mL of freshly prepared chloramine T in citrate buffer solution was subsequently added and reacted at 20 °C for 20 min. Then, 0.2 mL of perchloric acid was added to quench the reaction; 20% solution of P-dimethylaminobenzaldehyde (DMAB) in isopropanol was added to the solution and reacted at 60 °C for 20 min. The absorbance of the resulting solution at wavelength of 555 nm was read on Cary 50 UV–Vis spectrophotometer (Agilent Technologies, Santa Clara, CA, USA) against a hydroxyproline standard.

### 5.6. Gel Electrophoresis

Molecular weight distribution of peptides in the collected supernatants was determined by sodium dodecyl sulfate polyacrylamide gel electrophoresis (SDS–PAGE) following Laemli’s protocol [33]. Aliquoted supernatant samples were treated using a loading buffer containing 10% (*w/v*) SDS, 50% (*v/v*) glycerol, 500 mM DTT, 0.25 M Tris-HCl, pH 6.8, and 0.05% (*w/v*) bromophenol blue and subsequently loaded into the gel wells. Peptide separation was performed at a constant voltage of 120 V on 7% or 12% acrylamide gels. Pre-stained protein marker (Bio-Rad Laboratories, Hercules, CA, USA) was used as the molecular weight standard.

### 5.7. Size Exclusion Chromatography

Supernatant samples were analyzed on a Phenomenex BioSep-SEC-S2000 (300 mm × 7.8 mm). The column was calibrated using 180 to 670,000 g/mol standards. Sample injection volume was 20 µL and the elution was carried out in 50 mM sodium phosphate, 150 mM sodium chloride and pH 7.6. The flow rate was 0.5 mL/min with a total run time of 40 min. The eluted peaks were detected at 210 nm.

## Figures and Tables

**Table 1:** Swelling ratio of collagen hydrogel.

Type of Collagen Hydrogel	Swelling Ratio
Intact Collagen (C-coll)	724%
Hydrolyzed Collagen (D-coll)	778%

## Supplementary Materials

Supplementary materials can be found at https://www.mdpi.com/2310-2861/6/4/46/s1. Figure S1: Chromatograms of size-exclusion chromatography (SEC) of acetic acid.

## Abbreviations

C-coll	Intact collagen
CB	C-coll in Tris buffer
CC	C-coll in collagenase solution in Tris buffer
D-coll	Hydrolyzed collagen
DB	D-coll in Tris buffer
DC	D-coll in collagenase solution in Tris buffer
MDPI	Multidisciplinary Digital Publishing Institute
SEC	Size-exclusion column
SDS–PAGE	Sodium dodecyl sulfate–polyacrylamide gel electrophoresis
HHP	High molecular weight hydrolyzed peptides
LHP	Low molecular weight hydrolyzed peptides
