# Supplementary material for: Monitoring the Degradation of Collagen Hydrogels by Collagenase Clostridium histolyticum
Source: Gels. 2020 Nov 27;6(4):46. doi: 10.3390/gels6040046 (PMC7709630; doi:10.3390/gels6040046)
Supplement: Supplementary file 1 [file gels-06-00046-s001.pdf]

gels

Communication

Monitoring the Degradation of Collagen Hydrogels by Collagenase *Clostridium histolyticum*

Hon Wei Ng, Yi Zhang, Rafea Naffa and Sujay Prabakar *

Leather and Shoe Research Association of New Zealand, P.O. Box 8094, Palmerston North 4472, New Zealand
* Correspondence: sujay.prabakar@lasra.co.nz; Tel.: +64-6-355-9028

Received: 21 October 2020; Accepted: 24 November 2020; Published: 30 November 2020

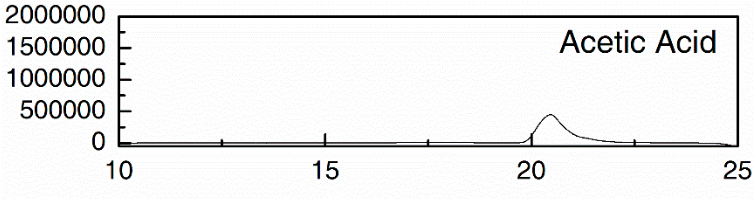

Figure S1. Chromatograms of size-exclusion chromatography (SEC) of acetic acid.

© 2020 by the authors. Licensee MDPI, Basel, Switzerland. This article is an open access article distributed under the terms and conditions of the Creative Commons Attribution (CC BY) license (http://creativecommons.org/licenses/by/4.0/).
